# CRISPR-Cas systems in the marine actinomycete *Salinispora*: linkages with phage defense, microdiversity and biogeography

**DOI:** 10.1186/1471-2164-15-936

**Published:** 2014-10-25

**Authors:** Matthias Wietz, Natalie Millán-Aguiñaga, Paul R Jensen

**Affiliations:** Scripps Institution of Oceanography, University of California San Diego, La Jolla, CA 92037 USA; Institute for Chemistry and Biology of the Marine Environment, University of Oldenburg, 26129 Oldenburg, Germany

**Keywords:** *Salinispora*, CRISPR-Cas, Prophages, Mobile genetic elements, Immunity, Evolution

## Abstract

**Background:**

Prokaryotic CRISPR-Cas systems confer resistance to viral infection and thus mediate bacteria-phage interactions. However, the distribution and functional diversity of CRISPRs among environmental bacteria remains largely unknown. Here, comparative genomics of 75 *Salinispora* strains provided insight into the diversity and distribution of CRISPR-Cas systems in a cosmopolitan marine actinomycete genus.

**Results:**

CRISPRs were found in all *Salinispora* strains, with the majority containing multiple loci and different Cas array subtypes. Of the six subtypes identified, three have not been previously described. A lower prophage frequency in *S. arenicola* was associated with a higher fraction of spacers matching *Salinispora* prophages compared to *S. tropica*, suggesting differing defensive capacities between *Salinispora* species. The occurrence of related prophages in strains from distant locations, as well as spacers matching those prophages inserted throughout spacer arrays, indicate recurring encounters with widely distributed phages over time. Linkages of CRISPR features with *Salinispora* microdiversity pointed to subclade-specific contacts with mobile genetic elements (MGEs). This included lineage-specific spacer deletions or insertions, which may reflect weak selective pressures to maintain immunity or distinct temporal interactions with MGEs, respectively. Biogeographic patterns in spacer and prophage distributions support the concept that *Salinispora* spp. encounter localized MGEs. Moreover, the presence of spacers matching housekeeping genes suggests that CRISPRs may have functions outside of viral defense.

**Conclusions:**

This study provides a comprehensive examination of CRISPR-Cas systems in a broadly distributed group of environmental bacteria. The ubiquity and diversity of CRISPRs in *Salinispora* suggests that CRISPR-mediated interactions with MGEs represent a major force in the ecology and evolution of this cosmopolitan marine actinomycete genus.

**Electronic supplementary material:**

The online version of this article (doi:10.1186/1471-2164-15-936) contains supplementary material, which is available to authorized users.

## Background

CRISPRs (clustered regularly interspaced short palindromic repeats) have been detected in approximately 85% of archaeal and 50% of bacterial genomes [1]. They are considered a means of prokaryotic adaptive immunity against bacteriophages [2], which are major determinants of prokaryotic abundance, diversity and community structure [3]. CRISPRs consist of conserved repeats separated by variable spacers, the latter representing incorporated fragments of viral or plasmid DNA that specify immunity upon subsequent encounters [4]. Many CRISPRs are associated with Cas gene arrays, which can be classified into three major types and ten subtypes [5, 6] and are considered essential for CRISPR function. The activity of CRISPR-Cas systems proceeds in three stages: the acquisition of protospacer sequences from foreign genetic elements and their integration into the CRISPR array, constitutive transcription of the array, and target interference through transcribed crRNA [2]. In response, phages have developed mechanisms to evade CRISPR action [7–9], suggesting a co-evolutionary arms race between bacteria and phages.

Comparative genomics has given insight into CRISPRs from *Actinobacteria* [10, 11], *Firmicutes* [12, 13], *Cyanobacteria* [14, 15], enterobacteria [16], and *Archaea* [17]. In addition, mathematical modeling has presented important concepts about CRISPR dynamics during phage-bacteria interactions [18, 19]. Most of what is known about CRISPRs has been derived from pathogenic or industrially relevant bacteria such as *Salmonella* [20] and *Streptococcus* [12]. In the case of environmental bacteria, it has been shown that CRISPRs are widespread in *Cyanobacteria* except for the major marine lineages *Prochlorococcus* and *Synechococcus* [15]. In freshwater *Cyanobacteria*, CRISPRs were used to illustrate specific host-cyanophage interactions [14]. Furthermore, CRISPRs have been linked to host-phage co-evolution, community structuring and biogeographic patterns in microbial mats [21], acidophilic biofilms [22], and hot spring microbiota [23].

CRISPRs also control genetic exchange [24, 25] and intraspecies recombination [26], hence mediating evolutionary processes [27]. They may also regulate gene expression via crRNAs [28] and ‘self-targeting spacers’ that match elements in the host genome [29]. CRISPR activity has also been linked to DNA repair [30] and can affect various bacterial phenotypes including biofilm formation [31], swarming motility [32], and pathogenicity [33]. Despite the insights afforded by these studies, the distribution, diversity and functional roles of CRISPR-Cas systems among closely related environmental bacteria remain largely unknown.

In the present study, we analyzed CRISPR-Cas and prophage content in 75 *Salinispora* strains from seven global collection sites. This actinomycete genus has a pan-tropical distribution in marine sediments [34, 35] and is comprised of three closely related species; the cosmopolitan *S. arenicola* and the regionally confined sister taxa *S. pacifica* and *S. tropica* [36, 37]. The species have been further divided into 16S rRNA phylotypes (i.e. single nucleotide variants), with the highest diversity in *S. pacifica* and the lowest in *S. tropica* [35] *.* The genus is recognized for the production of diverse secondary metabolites [38], with the associated biosynthetic pathways showing evidence of extensive horizontal gene transfer [39, 40].

The diversity and distribution of CRISPR-Cas systems in *Salinispora* spp. was investigated to (i) assess the role of CRISPRs in phage defense, (ii) characterize past interactions with foreign genetic elements, (iii) elucidate linkages between CRISPR features and *Salinispora* microdiversity, and (iv) identify biogeographic signatures in CRISPR and prophage content. The detected diversity of CRISPR-Cas systems, including spacers that match foreign genetic elements, supports a role in host immunity. Spacer arrays illustrated recurring encounters with related phages as well as geographically confined MGEs. These findings suggest the presence of complex CRISPR-mediated interactions between *Salinispora* spp. and foreign genetic elements that may influence the ecology and evolution of this broadly distributed marine actinomycete genus.

## Results and discussion

### CRISPR content in 75 *Salinispora*strains

Genome sequences from 75 *Salinispora* strains derived from seven global collection sites were analyzed for CRISPR-Cas content (Additional file 1). In total, 335 CRISPR loci were detected, with an average of 4.4 per strain (Table 1) but considerable among strain variability, ranging between 1 and 12 (Additional file 1). Unlike many genera for which multiple genome sequences are available, all 75 *Salinispora* strains harbored CRISPRs, suggesting they are an ecologically relevant feature of this genus. *Salinispora* CRISPR content exceeded the average reported for mesophilic bacteria [1] and marine bacterial metagenomes [41] and accounted for up to 0.3% of some genomes, which is approximately a third of the reported ‘prokaryotic maximum’ [2]. CRISPRs were concentrated in genomic islands, which represent the major regions of gene acquisition in *Salinispora* spp. [40]. Prior evidence of extensive horizontal gene transfer in *Salinispora* spp. [39, 40], coupled with the ubiquity of CRISPR-Cas systems detected in the present study, suggests an ongoing dynamic between CRISPR-mediated immunity and the acquisition of foreign genetic material.

**Table 1:** **Summary of CRISPR-Cas and prophage content in** ***Salinispora*** **spp.**

Species	Genomes analyzed	Total CRISPRs	Avg. loci/strain (per Mb)	Avg. locus size ± SD (bp)	Loci with Cas arrays (%)	Total spacers	Avg. spacers/ strain (±SD)	Avg. prophages/strain	Spacers matching ***Salinispora***prophages/known MGEs (%)*
*S. arenicola*	37	169	4.5 (0.8)	1243 ± 1087	78 (56)	3033	82 ± 52	1.3	18.3/2.5
*S. pacifica*	31	136	4.4 (0.8)	1110 ± 1087	54 (63)	2153	69 ± 42	1.1	8.9/0.6
*S. tropica*	7	30	4.3 (0.8)	1362 ± 1086	14 (63)	551	79 ± 57	2.1	4.5/0.2

*only considering perfect matches (100% sequence identity/coverage).

The 335 CRISPR loci contained 5737 spacers, of which 68% were observed only once across all genomes. Extensive differences in spacer content were detected among strains isolated at the same time from the same site (Additional file 1), suggesting that spatiotemporal encounters with mobile genetic elements (MGEs) may be highly variable. On average, *S. arenicola* and *S. tropica* contained more spacers per strain than *S. pacifica*, however, the numbers varied greatly among strains within each species (Table 1).

### Diversity and evolution of Cas array subtypes

**Figure 1:**
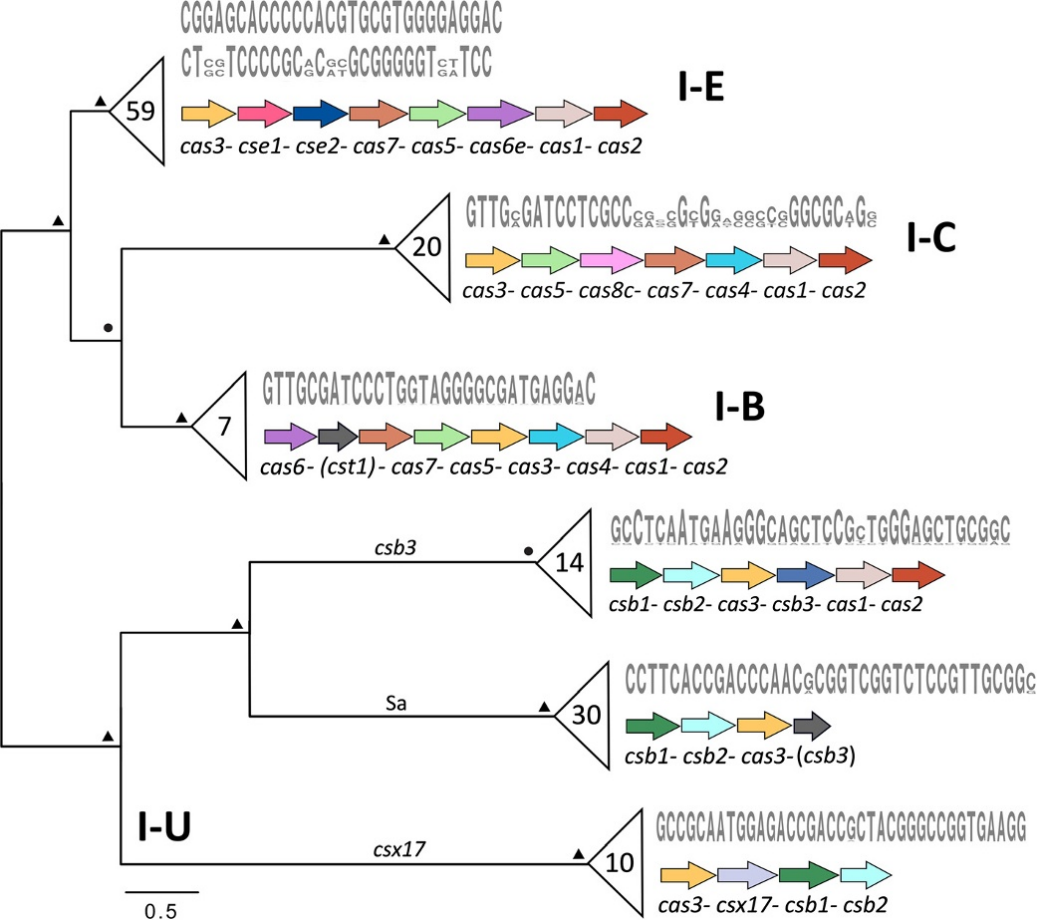
***cas3*** **phylogeny and CRISPR repeat diversity.** Condensed maximum likelihood phylogeny of *cas3* nucleotide sequences reveals clades corresponding to Cas array subtype. The two major clades delineate known **(I-E, I-C, I-B)** and previously undescribed **(I-U)** subtypes. The order of genes for each subtype is displayed on the right. Gene annotations in parentheses designate hypothetical proteins with low identity to those indicated. In the I-B arrays, *cas8b* was replaced by a larger gene related to *cst1*. The total number of each array subtype among the 75 genomes is shown in the condensed nodes. Six arrays were missing several genes and therefore excluded from the analysis. Nodal support values (● above 80%, ▲ 100%) were obtained by 1000 bootstrap replicates (see Additional file 2 for the full tree including strain names and bootstrap values). Consensus repeat sequences in the associated CRISPR loci (indicated in gray) were specific to each array subtype and mostly showed considerable conservation.

The 75 *Salinispora* strains contained 146 Cas arrays (Table 1), all of which can be classified as type I based on the inclusion of a *cas3* gene [6]. Cas arrays could be further grouped into six subtypes (Figure 1), of which five occurred in all three species and one (I-U_Sa) was only observed in *S. arenicola*. In total, 60% of the CRISPRs were associated with Cas arrays (Table 1), with up to five different array subtypes in some strains (Additional file 1). Three of these subtypes (I-E, I-C, I-B) have been previously characterized [6], with the most common (I-E) occurring in 49 strains. Almost two-thirds of the I-E arrays were associated with paired loci, i.e., two CRISPRs (one with inverted repeat sequences) flanking internalized *cas* genes, as often observed in *Archaea* [42]. Eleven strains contained two I-E or I-C arrays (Additional file 1). BLAST analysis of the associated *cas3* genes indicated that the two arrays in a given strain were acquired as independent events from different sources based on sequence similarities to homologs in different actinomycetes (*Verrucosispora* vs. *Streptomyces* spp. for I-E arrays and *Frankia* vs. *Stackebrandtia* spp. for I-C arrays). In all three species, the GC content of I-C arrays was lower than the overall genomic GC content. This was especially apparent in *S. pacifica* (64.2% vs. 69.8% GC), suggesting that I-C arrays have been acquired from distantly related taxa. To the best of our knowledge, three of the Cas array subtypes detected (herein designated as I-U_csb3, I-U_csx17 and I-U_Sa) have not previously been described despite containing known *cas* genes (*csb1*, *csb2*, *csb3*, *csx17*). These subtypes were designated as I-U based on convention [6]. However, the Integrated Microbial Genomes (IMG) database [43] revealed that a variety of bacteria from different phylogenetic groups possess equivalent arrays, indicating these are not unique to *Salinispora* spp.

*cas3* is the signature gene of type I arrays [6]. A *cas3* phylogeny revealed clades that corresponded to Cas array subtype as opposed to taxonomic relationships (Figure 1). The finding of *cas3* sequence similarities across species boundaries supports the concept that Cas arrays evolve independent of their hosts [20, 44]. Furthermore, sequences within the array subtypes reveal evidence of recombination, as different *Salinispora* species shared virtually identical *cas3* genes. The same patterns were observed with *cas1* genes and corresponding protein sequences (Additional file 2), the most common phylogenetic marker for CRISPR-Cas systems. The delineation of the Cas array subtypes was supported by the repeat sequences, which frequently shared subtype-specific conservation (Figure 1) and averaged between 29 nt (subtypes I-E and I-B) and 37 nt (subtypes I-C and I-U).

Cas-associated CRISPRs contained significantly more spacers than Cas-devoid loci (*p* < 1 × 10^-10^), as might be expected given that *cas* genes are required for spacer integration [2]. Furthermore, subtypes I-E, I-C and I-B contained significantly more spacers (*p* < 0.0001) than the three I-U subtypes. Considering the latter, I-U_Sa and I-U_csx17 lack *cas1* and are thus potentially unable to incorporate additional spacers, as *cas1* is involved in spacer integration [2].

### CRISPRs illustrate interactions with foreign genetic elements

We assessed defensive functions of *Salinispora* CRISPRs by analyzing for perfect matches between *Salinispora* spacers and mobile genetic elements (MGEs). These included 97 prophages that were identified in the 75 genomes (Additional file 3) as well as MGEs deposited in the Aclame database [45] (the latter referred to as ‘known MGEs’). On average, 11% of spacers matched *Salinispora* prophages (Table 1). Prophage-devoid strains had a higher fraction of matching spacers than prophage-harboring strains (*p* < 0.05), supporting a functional role of CRISPRs in phage immunity. In addition, 1.1% of spacers matched known MGEs, which was comparable to observations for marine bacterial metagenomes [41] and oral pathogens [26]. Some spacers matched homologous elements from different viral genomes, suggesting they may target multiple phage strains. CRISPRTarget [46] revealed that MGEs matched by *Salinispora* spacers are associated with various protospacer-associated motifs (PAMs), short sequences important for protospacer acquisition [2]. This suggests that *Salinispora* spp. can detect different PAMs and integrate a large diversity of spacers. When including lower-quality matches (100% identity over at least 18 nt) the majority (77%) of spacers matched plasmids, suggesting that a major role for *Salinispora* CRISPRs is to defend against plasmid integration. As no information about the plasmid content of the strains investigated is currently available, we focused on the role of CRISPRs in phage defense, while realizing this may not present a complete picture of CRISPR functionality in *Salinispora* spp.

### CRISPRs indicate differing defensive capacities among *Salinispora*species

*S. arenicola* had four-fold more spacers matching *Salinispora* prophages and twelve-fold more spacers matching known MGEs than *S. tropica*. This corresponded to the fact that only two-third of *S. arenicola* but all *S. tropica* strains harbored prophages, with 1.3 vs. 2.1 prophages per genome, respectively (Table 1). A substantial number of *S. arenicola* spacers that matched *Salinispora* prophages were located in the I-U_Sa Cas arrays, which are specific to *S. arenicola*. This additional array and spacer diversity may provide superior defensive capacities for *S. arenicola*, which potentially contributes to its broader geographic distribution [35]. *S. pacifica* had an intermediate fraction of spacers matching *Salinispora* prophages and known MGEs, with 1.1 prophages per genome (Table 1). There was a significantly lower frequency of prophages among phylotype C and F strains (*p* < 0.01). While these phylotypes also contained significantly more spacers (*p* < 0.01), the fraction of those spacers matching *Salinispora* prophages and known MGEs was similar to other phylotypes. The differing phage sensitivities between *S. pacifica* phylotypes are thus independent from or only partially related to CRISPRs.

In contrast, the total numbers of CRISPR loci or spacers were uncorrelated with prophage content in all three species (*R*^*2*^ < 0.01). For instance, strains CNS-051 and CNS-205 contained 11 and 8 CRISPRs with 119 and 140 spacers, respectively. Despite these similarities, these strains harbored 0 and 5 prophages, respectively (Additional file 1). The number and diversity of Cas arrays were also uncorrelated with prophage content (*R*^*2*^ < 0.001). For instance, *S. pacifica* strain DSM-45549 contained four Cas array subtypes and three prophages while the Cas-devoid *S. pacifica* strain CNS-103 only contained one prophage (Additional file 1). Thus, the number of CRISPR loci as well as the diversity of associated Cas arrays appear to be affected by factors other than phage exposure.

### History of *Salinispora*interactions with a common prophage

**Figure 2:**
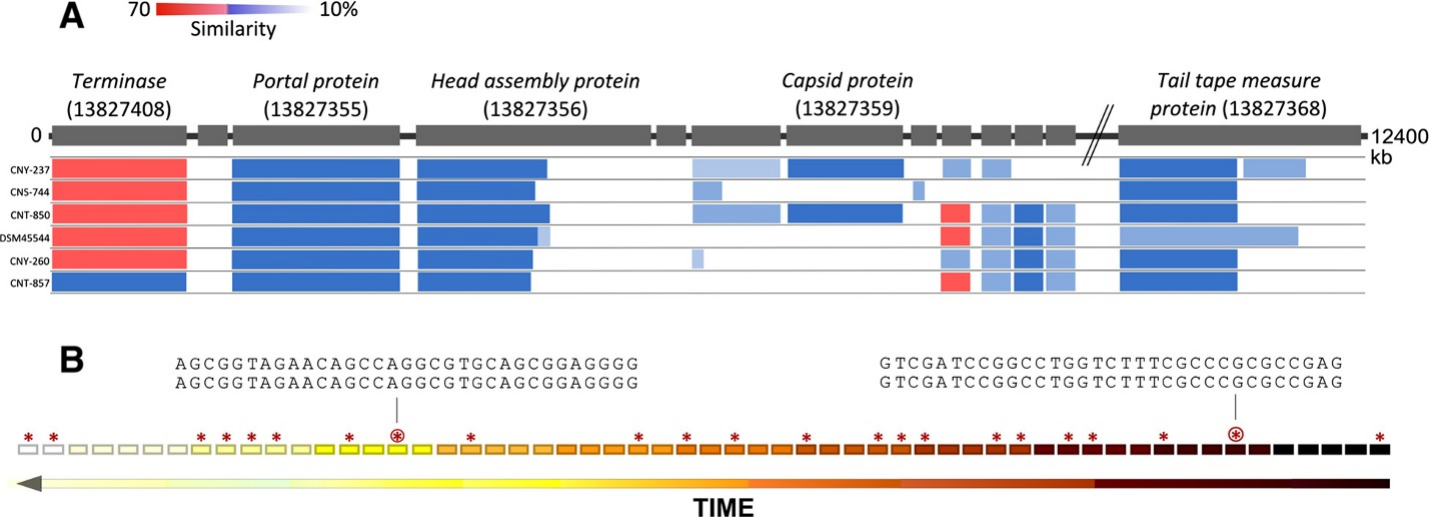
***Streptomyces*** **SV1-related prophage content in** ***Salinispora*** **spp. A)** Comparison of genes from six intact, SV1-related prophages in *Salinispora* genomes with the SV1 type phage (gray; with GenBank Gene IDs in parentheses) by color-coding percent sequence similarities over a 12400 bp region within SV1. **B)** History of SV1 encounters in *S. tropica* CNR-699 as reflected in multiple spacer matches in a single array. Incomplete matches are indicated by asterisks, perfect matches by encircled asterisks with sequences shown above.

We focused on a common prophage that is related to the *Streptomyces* SV1 phage and was detected in 24 *Salinispora* strains from all three species (Figure 2A, Additional file 3). Six percent of *Salinispora* spacers matched SV1-related sequences, suggesting that this phage represents a major challenge to the genus. Strains without an integrated SV1 prophage had a larger fraction of spacers matching SV1 in Cas-associated loci (89%) compared to those with an integrated SV1 prophage (75%), supporting a specific targeting of this phage. The history of encounters with SV1-related phages was determined for six strains per species (three harboring and three lacking SV1) by analyzing the location of matching spacers within spacer arrays according to the concept that ancestral spacers are commonly located at the ‘trailer’ end and more recent spacers at the ‘leader’ end of a spacer array [4]. Matching spacers, the majority with unique sequences, were detected throughout the spacer arrays (Figure 2B) suggesting recurring encounters with SV1-related phages over time. Given that the SV1 phage represents a vector for genetic exchange [47], it is interesting to speculate that it may represent a source of beneficial genetic material in addition to a survival challenge.

### Linkages of CRISPR-Cas features with microdiversity

*Salinispora* microdiversity on the subspecies level has been defined based on 16S rRNA phylotypes (Additional file 1) and a multilocus phylogeny (Additional file 4). We detected several correlations between CRISPR-Cas features and microdiversity. For instance, one well-supported *S. pacifica* lineage contained the only strains (CNT-796 and CNT-851) with a modified I-C array lacking *cas1*/*cas2*, suggesting these genes have been lost in this lineage. Another *S. pacifica* lineage (containing strains CNQ-768 and CNS-103) was unique in being entirely devoid of *cas* genes. Also, certain clades were characterized by chromosomal relocations of CRISPR-Cas systems, as seen with I-E arrays in *S. pacifica* (strains CNT-796 and CNT-851) and *S. tropica* (strains CNS-197 and CNR-699).

**Figure 3:**
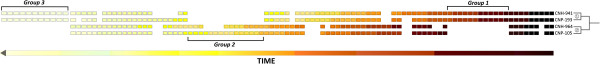
**Subclade-specific spacer content in** ***S. arenicola*** **phylotype B.** Alignment of spacers from a homologous CRISPR locus present in all four *S. arenicola* phylotype B strains. The phylogenetic relationships among the four strains are depicted on the right. Spacers are indicated by rectangles arranged from the oldest (trailer end; right) to the most recent spacers (leader end; left). Vertically aligned spacers are identical and separated by subclade-specific groups of spacers (1, 2, 3), which were likely deleted from the respective other lineage or selectively acquired.

The most distinct linkages were observed among the four *S. arenicola* phylotype B strains, which contained significantly more CRISPRs and spacers than strains from *S. arenicola* phylotypes A and ST (*p* < 0.05). Many spacers were unique to phylotype B, underlining that spacer composition can reflect population structure and evolutionary relationships [48, 49]. CRISPR characteristics not only distinguished phylotype B from other phylotypes, but also the two subclades within phylotype B (strains CNH-941 and CNP-193 vs. CNH-964 and CNP-105; Additional file 4). For instance, a paired CRISPR locus and flanking genes were inverted in one of the subclades (Additional file 5). Furthermore, there were subclade-specific differences in spacer content. While multiple spacers were shared by all phylotype B strains, which is consistent with observations among other closely related bacteria [46], spacer array alignments revealed three sets of spacers that were specific to one of the subclades (Figure 3). This probably illustrates subclade-specific deletions or insertions of whole spacer groups [49]. Sixty-five percent of the group 1 spacers in CNH-941 and CNP-193 matched plasmids from Alphaproteobacteria, while the group 2 spacers in CNH-964 and CNP-105 equally matched phages and largely gammaproteobacterial plasmids. This may coincide with differing defensive capacities or varying modes of interaction with MGEs between the two subclades. While prophage content appeared independent of these observations (Additional file 2) MGEs are also involved in diversification [50, 51], niche adaptation [52], and microdiversity [53]. It is hence interesting to speculate that these differences may influence the evolutionary or ecological divergence within *S. arenicola* phylotype B.

### Biogeographic patterns in CRISPR and prophage content

**Figure 4:**
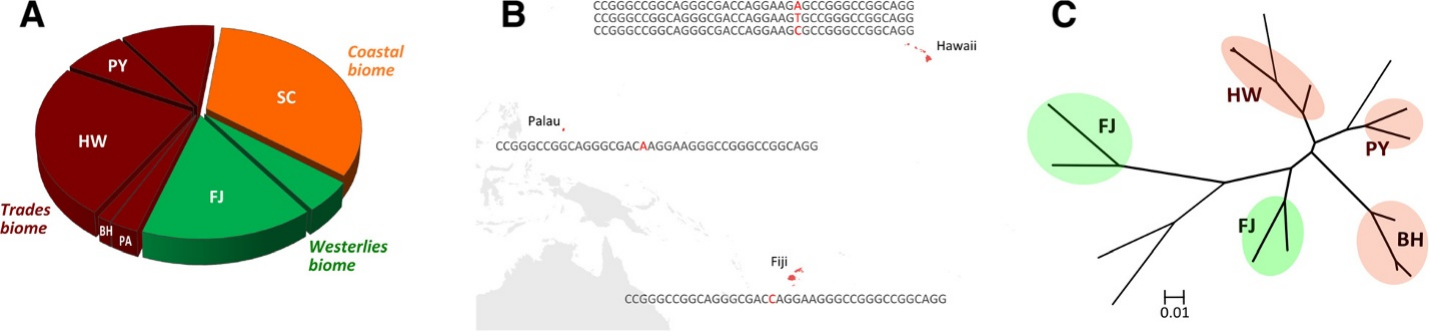
**Biogeographic patterns among** ***Salinispora*** **spacers and prophages. A)** Distribution of spacers unique to certain locations (SC = Sea of Cortez, HW = Hawaii, FJ = Fiji, PY = Palmyra, BH = Bahamas, PA = Palau) and biomes; **B)** Location-specific spacer variants reflected in single nucleotide polymorphisms (marked in red) within a conserved 41 nt spacer in strains from Hawaii (3 strains with 3 variants), Fiji (4 strains with 1 variant) and Palau (1 strains with 1 variant); **C)** Maximum likelihood phylogeny (1000 bootstrap replicates) of SV1-related prophages in genomes from geographically distant *Salinispora* strains, showing location-specific SV1 lineages.

The strains analyzed in this study originate from seven global collection sites and were derived from independent sediment samples. While sampling efforts were not uniform across locations and may have affected the biogeographic patterns observed, it is interesting to note that 40% of the spacers observed in more than one strain were restricted to specific locations and/or biomes, the latter describing major oceanic regions distinguished by oceanographic factors such as nutrient concentrations and primary productivity [54]. Location-specific spacers provide evidence of exposure to local virus populations [41, 55], with the majority of localized spacers occurring in strains from the Sea of Cortez (Figure 4A). This is a highly productive sea [56] enclosed by a distinct geographical barrier and the only site classified as a Coastal biome [54]. While these results are preliminary, it is intriguing to speculate that spacer sequences can be used to trace location-specific interactions with distinct MGE pools, as also observed in other ecosystems [23, 57, 58]. A more nuanced biogeographic pattern was the detection of identical spacers with location-specific nucleotide substitutions, as found in strains from Hawaii, Fiji and Palau (Figure 4B). This may illustrate the presence of widespread MGEs that maintain location-specific genetic variants. Furthermore, SV1-related prophages could be resolved into geographically confined lineages (Figure 4C), supporting the concept that *Salinispora* strains are exposed to location-specific MGEs.

### Self-targeting spacers

Several studies have reported the occurrence of ‘self-targeting spacers’ that match regions within the host genome [29]. While self-targeting spacers can be deleterious and strongly selected against [29, 59], they have also been suggested to function as regulatory elements [20, 60, 61] or to affect genome content [62]. Interestingly, a third of the 75 *Salinispora* strains harbored such spacers, with perfect matches to e.g. a cytochrome P450 within a terpenoid biosynthetic pathway [40] and two DNA-modifying genes (Table 2). However, experimental evidence would be required to determine potential regulatory roles. In addition, several self-targeting spacers matched resident prophages, suggesting that CRISPR interference may be ineffective in some cases. Alternatively, self-targeting may be prevented by selective self vs. non-self mechanisms, such as variations in spacer flanking sequences [63].

**Table 2:** **Chromosomal matches of select self-targeting spacers**

Strain	Spacer match (IMG Gene ID)	Spacer sequence (above) and matching chromosomal region with adjacent nucleotides (5′-3′; below)
*S. arenicola* CNH-964/*S. arenicola* CNP-105	Adenylosuccinate lyase (2515702456/2518452715)	GCCCACCTTGCCGTGCCACCACGCCTCCCGCACCTCGTT
		GTGCTGCTGCCCACCTTGCCGTGCCACCACGCCTCCCGCACCTCGTTGAACTCCG
	23S rRNA methyltransferase (2515702034/2518452486)	CCGAGCGGGTCGAGCTGACCGTCGGGGCGGTGGCCCCGGG
		GCGGAGGCCGAGCGGGTCGAGCTGACCGTCGGGGCGGTGGCCCCGGGCGGGCAC
*S. arenicola* CNX-481	Cytochrome P450 (2518471737)	TACCGACGCAGCCATAACTCGTGCTAGGACGG
		CTGATGGCTACCGACGCAGCCATAACTCGTGCTAGGACGGTGCCCGCG

## Conclusions

This study describes a comprehensive survey of CRISPR-Cas systems among a large collection of strains from a cosmopolitan marine actinomycete genus. The finding of ubiquitous and diverse CRISPR-Cas systems suggests that *Salinispora* maintains a robust mechanism to mediate interactions with MGEs, which may be of ecological and evolutionary relevance in virally rich marine sediments [3]. Future surveys of CRISPR-Cas systems will provide additional opportunities to assess the evolutionary history of MGE exposure, the effectiveness of these systems as mechanisms of adaptive defense, and how CRISPRs may be linked to the ecology and evolution of *Salinispora*.

## Methods

### Genome sequences and CRISPR-Cas classification

The 75 *Salinispora* genome sequences (Additional file 1) were downloaded from the IMG database (https://img.jgi.doe.gov). CRISPRs were predicted using CRISPRFinder [64] on pseudochromosomes generated from the genome sequences (i.e. contigs assembled using a closed reference genome) [39] and unmapped contigs. Only CRISPRs classified as ‘confirmed’ were considered. Predicted CRISPRs were manually checked and adjacent loci combined if separated by Ns and having the same repeat sequences. Annotated *cas* genes were verified by determining similarities to known *cas* genes using BLAST [65] and UniProt [66]. The naming of *cas* genes and their classification into Cas array subtypes was done following [6]. The IMG database was searched for equivalent Cas arrays in other sequenced bacterial genomes. CRISPRmap was used to classify repeats into motifs, families, and superclasses based on similarities to known repeat sequences [67]. Repeat consensus sequences were obtained using WebLogo [68].

### Phylogenetic and structural analyses of Cas arrays

*cas1* and *cas3* nucleotide and corresponding Cas1 and Cas3 amino acid sequences were aligned using MAFFT v7.017 (L-INS-i algorithm, 100PAM/k = 2 scoring matrix, gap open penalty 1.53, offset value 0.123) [69] and manually curated. The best substitution models (*cas1*: TN93 + G + I; Cas1: WAG + G + F; *cas3*: T92 + G; Cas3: JTT + G) were determined using MEGA5 [70]. Maximum likelihood phylogenies were computed with MEGA5 (using the best model and 100 bootstrap replicates) and RAxML (with default settings and 1000 bootstrap replicates) implemented on the CIPRES Science Portal [71], always giving the same topology. Nucleotide sequences of *cas1* [KM526976-KM527070] and *cas3* [KJ677987-KJ678124] have been deposited at GenBank (Additional file 6). Architectures of selected loci and flanking regions were analyzed with progressiveMauve [72]. Spacer arrangement in *S. arenicola* phylotype B was evaluated by aligning concatenated spacer sequences (sorted from trailer to leader end) with MAFFT [69].

### Prophage prediction and sequence comparison

Prophages were predicted using PHAST [73] on both the pseudochromosomes and unmapped contigs. Predicted intact prophages classified as being related to the *Streptomyces* SV1 phage were compared with the sequenced SV1 type phage (GenBank accession number NC_018848) using the CGView Comparison Tool [74]. Nucleotide sequences of SV1-related prophages were aligned using Mugsy [75] and the resulting alignment file converted to Fasta using the Galaxy web server [76]. The alignment was manually curated and the best substitution model (GTR + G) determined using MEGA5 [70]. A maximum likelihood phylogeny was computed using MEGA5 with 1000 bootstrap replicates (Additional file 7).

### Analysis of spacers

Spacers were extracted from genome sequences and sorted by unique (only found once across all 75 genomes) and shared (found in ≥2 genomes). Spacers were searched against different databases (Aclame MGE_0.4, PHAST_virus, PHAST_prophage_virus, CRISPRFinder spacer) with the standard BLAST parameters for short query sequences (word size 7; match/mismatch scores 1,-3; gap costs 5,2) using Geneious Pro v5.5 (available from http://geneious.com). In addition, short-query BLAST was used to determine spacers matching *Salinispora* prophages as well as self-targeting spacers matching non-CRISPR regions. Furthermore, short-query BLAST against *Salinispora* prophages was done with spacers from five representative strains from each species that were sorted by Cas-associated, Cas-devoid, associated with known Cas array subtypes (I-E, I-C, I-B), and associated with herein designated Cas array subtypes (I-U). Only perfect matches with 100% identity over the entire spacer length were considered. A separate BLAST search against Aclame was performed which also considered incomplete hits (100% sequence identity over at least 18 nt), as this may still be indicative of the targeted MGE type. The 18 nt threshold corresponds to 2/3 of the average *Salinispora* repeat length, which has been suggested as the minimum for a functioning spacer [1]. Also, 100% coverage hits are possibly rare since the vast majority of phage diversity is likely still unknown [3].

### Statistical evaluation

The number of CRISPR loci, prophages and MGE genes per strain were normalized by genome size and gene count, respectively. Values were compared by species, location, and biome (both between and within species) as well as phylotype (only within species) using the Kruskal-Wallis one-way analysis of variance implemented in R [77] to test for significant differences. In case of a significant result (*p* < 0.05) the Wilcoxon rank-sum test implemented in R [77] was used to test the specific sample pairs for significant differences (*p* < 0.05). The fraction of spacers matching *Salinispora* prophages in strains with and without prophages was compared using Student’s *t*-test. Correlations between the number of CRISPRs/spacers/Cas arrays and prophages were calculated using least squares regression.

#### Availability of supporting data

All supporting data are included within the article and its additional files.

## Electronic supplementary material

Additional file 1: **Overview of genome and CRISPR features of 75** ***Salinispora*** **strains.** Origin (location, biome, latitude/longitude, sampling date, depth) and general genome characteristics (genome size, gene count, 16S rRNA phylotype), CRISPR content (number of loci and spacers, Cas array diversity, CRISPRmap classification of repeats), number of prophages, and MGE content of 75 *Salinispora* strains analyzed in the present study. (XLSX 32 KB)

Additional file 2: **Phylogeny of** ***cas*** **genes and Cas proteins.** Maximum likelihood phylogenies of aligned *cas1*/*cas3* nucleotide as well as Cas1/Cas3 amino acid sequences (1000 bootstrap replicates with only those >50 shown). Species names abbreviated (SA = *S. arenicola*, SP = *S. pacifica*, ST = *S. tropica*) followed by strain number, Cas array subtype, and internal CRISPR locus ID. (PNG 5 MB)

Additional file 3: **Detected prophages.** Prophages detected in the 75 *Salinispora* genomes, classified based on sequence similarities with known prophages [73], length in Kb, number of coding sequences (CDS) and GC content (% GC). (XLSX 21 KB)

Additional file 4: ***Salinispora*** **species phylogeny.** Maximum likelihood phylogeny (1000 bootstrap replicates) of ten single-copy, concatenated housekeeping genes from 75 *Salinispora* genomes labeled with origin and phylotype (ST; A-F). A detailed description can be found in the original publication [39]. (PNG 715 KB)

Additional file 5: **Subclade-specific architectures of CRISPR loci and flanking genes.** progressiveMauve alignment of paired CRISPR loci and flanking genes in *S. arenicola* phylotype B, showing that the arrays are inverted in subclade 1 (strains CNH-941 and CNP-193) compared to subclade 2 (strains CNH-964 and CNP-105). Blue: CRISPRs, yellow: *cas* genes, green: integrases; pink: tRNAs. (PNG 138 KB)

Additional file 6: ***Salinispora cas*** **gene accession numbers.** GenBank accession numbers of *Salinispora cas1* and *cas3* sequences used for phylogenetic analyses. (XLSX 16 KB)

Additional file 7: **SV1 prophage phylogeny.** Maximum likelihood phylogeny (1000 bootstrap replicates) of conserved regions within SV1-related prophages in *Salinispora* genomes. (PNG 274 KB)
