# Supplementary figures and images for: CRISPR-Cas systems in the marine actinomycete Salinispora: linkages with phage defense, microdiversity and biogeography
Source: BMC Genomics. 2014 Oct 25;15(1):936. doi: 10.1186/1471-2164-15-936 (PMC4223832; doi:10.1186/1471-2164-15-936)

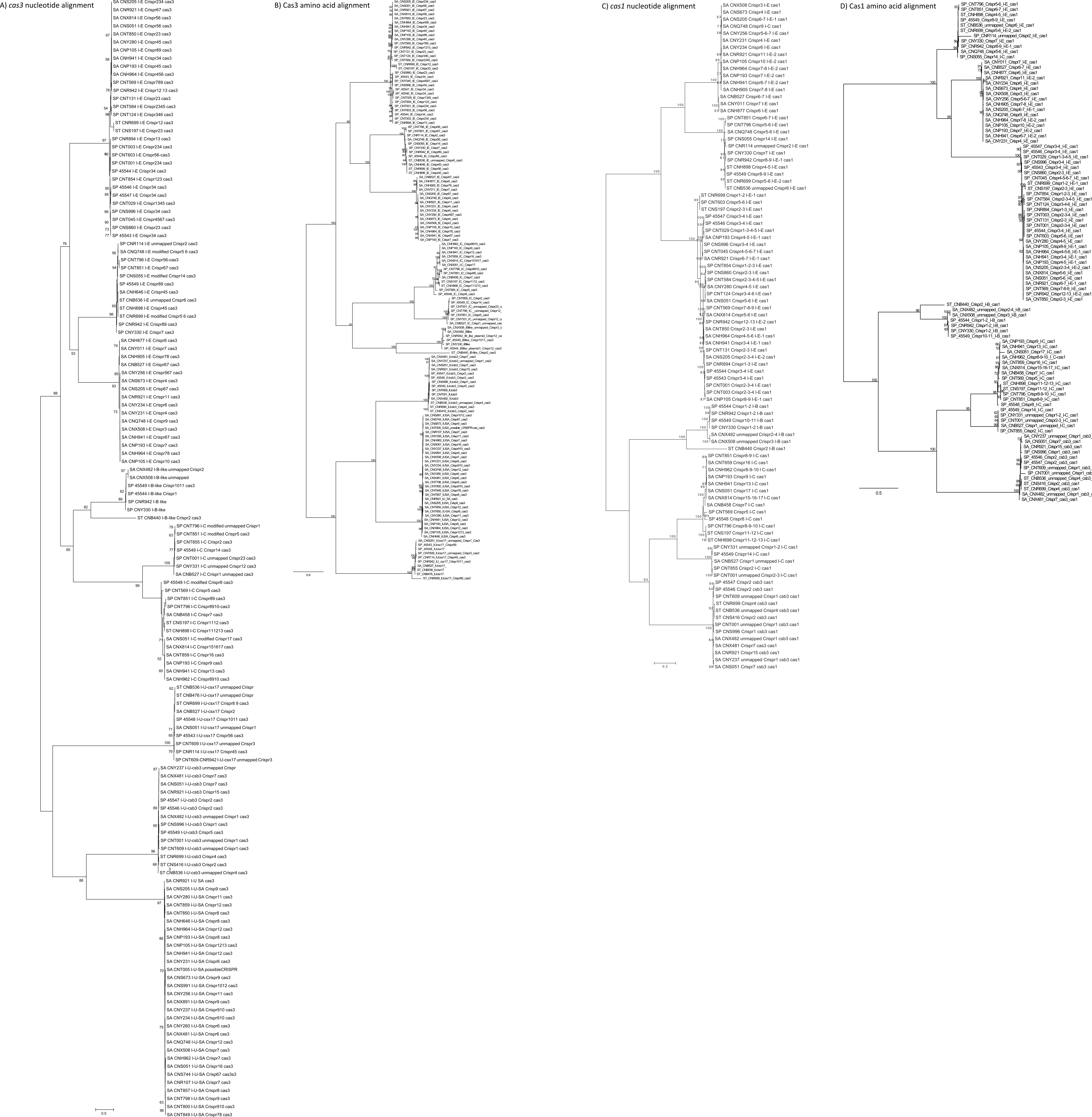

Supplement: Supplementary file 2 — Additional file 2: Phylogeny of cas genes and Cas proteins. Maximum likelihood phylogenies of aligned cas1/cas3 nucleotide as well as Cas1/Cas3 amino acid sequences (1000 bootstrap replicates with only those >50 shown). Species names abbreviated (SA = S. arenicola, SP = S. pacifica, ST = S. tropica) followed by strain number, Cas array subtype, and internal CRISPR locus ID. (PNG 5 MB) [file 12864_2014_6633_MOESM2_ESM.png]

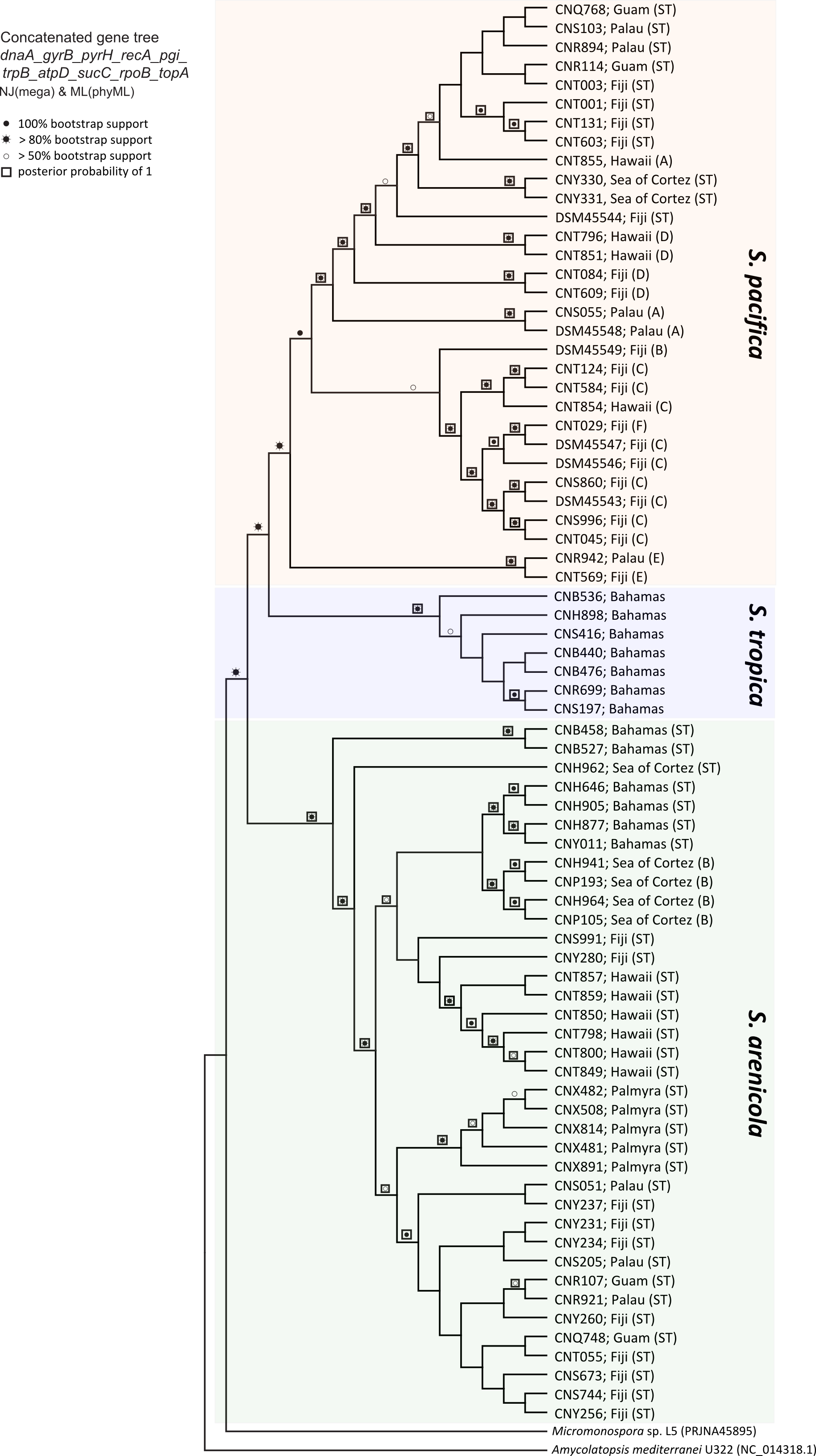

Supplement: Supplementary file 4 — Additional file 4: Salinispora species phylogeny. Maximum likelihood phylogeny (1000 bootstrap replicates) of ten single-copy, concatenated housekeeping genes from 75 Salinispora genomes labeled with origin and phylotype (ST; A-F). A detailed description can be found in the original publication [39]. (PNG 715 KB) [file 12864_2014_6633_MOESM4_ESM.png]

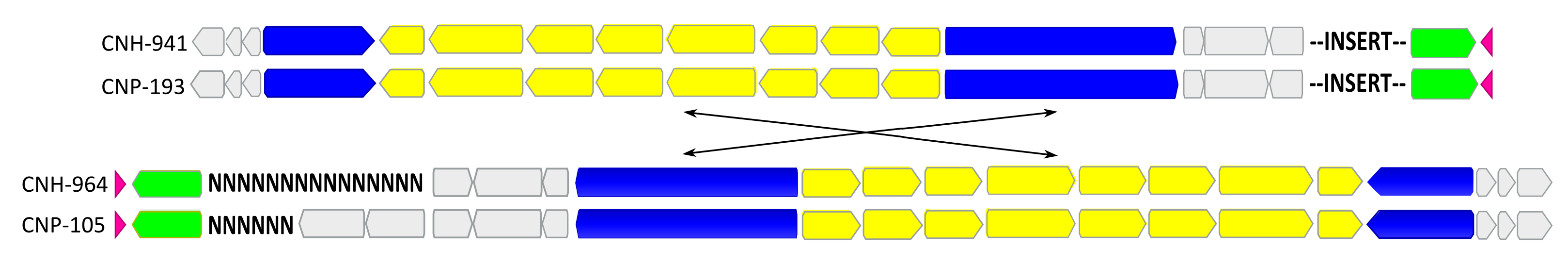

Supplement: Supplementary file 5 — Additional file 5: Subclade-specific architectures of CRISPR loci and flanking genes. progressiveMauve alignment of paired CRISPR loci and flanking genes in S. arenicola phylotype B, showing that the arrays are inverted in subclade 1 (strains CNH-941 and CNP-193) compared to subclade 2 (strains CNH-964 and CNP-105). Blue: CRISPRs, yellow: cas genes, green: integrases; pink: tRNAs. (PNG 138 KB) [file 12864_2014_6633_MOESM5_ESM.png]

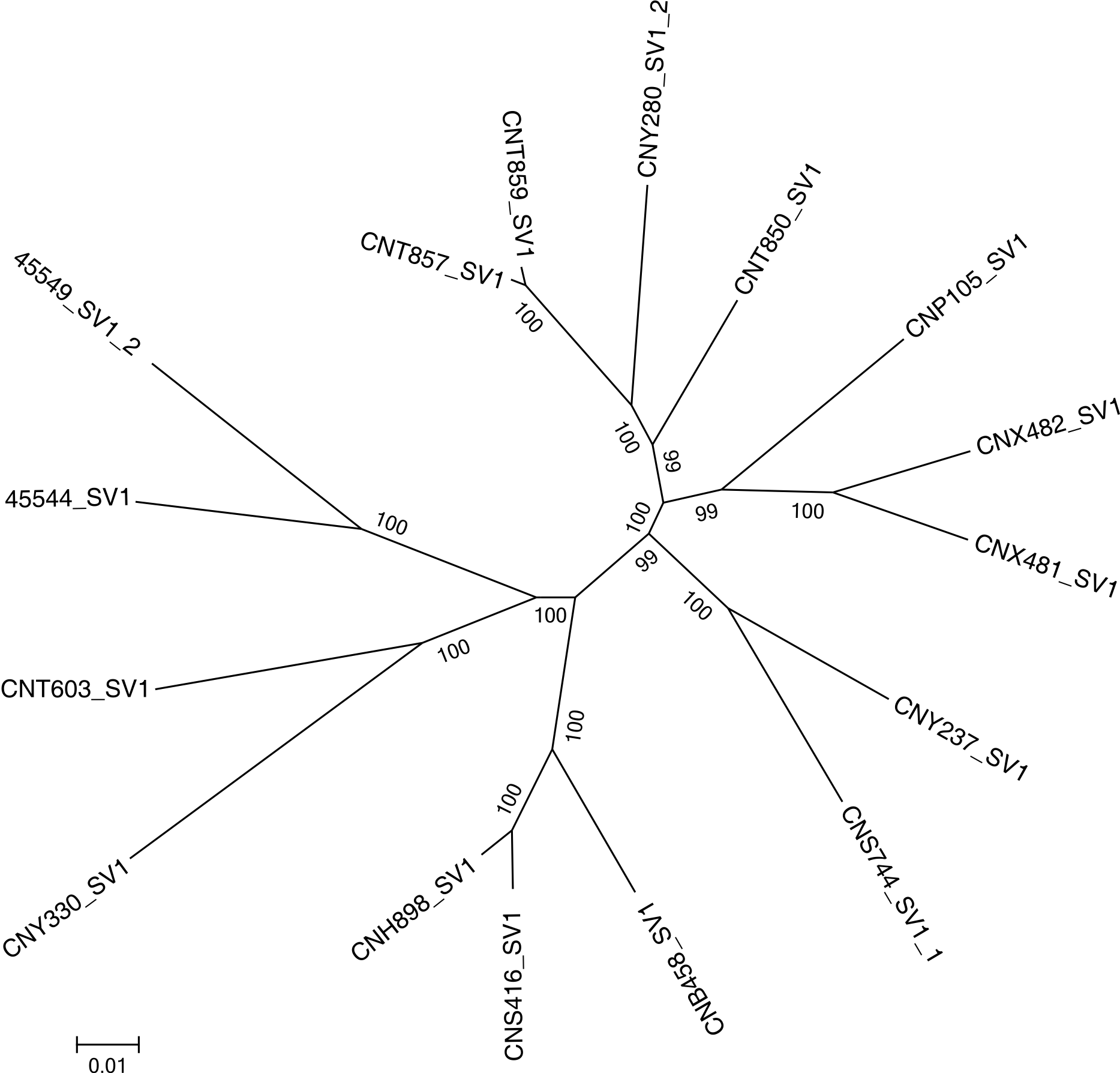

Supplement: Supplementary file 7 — Additional file 7: SV1 prophage phylogeny. Maximum likelihood phylogeny (1000 bootstrap replicates) of conserved regions within SV1-related prophages in Salinispora genomes. (PNG 274 KB) [file 12864_2014_6633_MOESM7_ESM.png]
